# Broad-Spectrum Hepatoprotection by *Pteropyrum scoparium* Extract Against Multi-Pesticide Oxidative Stress in Rats

**DOI:** 10.3390/foods15071123

**Published:** 2026-03-24

**Authors:** Amal M. Al-Nasiri, Mostafa I. Waly, Ahmed Al-Alawi, Lyutha Al-Subhi, Haytham Ali, Khalid Al Zuhaibi

**Affiliations:** 1 Department of Food Science and Nutrition, College of Agricultural and Marine Sciences, Sultan Qaboos University, Muscat P.O. Box 34, Oman; ahmed543@squ.edu.om (A.A.-A.); lyutha@squ.edu.om (L.A.-S.); 2 Department of Animal and Veterinary Science, College of Agricultural and Marine Sciences, Sultan Qaboos University, Al-Khoud, Muscat P.O. Box 34, Oman; h.ali@squ.edu.om; 3 Food Safety and Quality Centre, Ministry of Agriculture, Fisheries and Water Resources, Muscat P.O. Box 467, Oman; khalid771.km@gmail.com

**Keywords:** oxidative stress, pesticides, *Pteropyrum scoparium* extract, rat liver tissue, natural products

## Abstract

Chronic exposure to even low levels of pesticides is a serious public health issue, mainly due to the role of oxidative stress in damaging the liver and promoting cancer. This has driven interest in finding natural, plant-based antioxidants that can counteract this kind of chemical injury. In this study, we tested whether a methanol extract from the leaves of *Pteropyrum scoparium* (PSE) could protect the liver against oxidative harm caused by four common pesticides: acetochlor, deltamethrin, thiamethoxam, and rotenone. Chemical analysis showed that the extract contains high levels of phenolics (345.1 ± 7.6 mg GAE/g) and flavonoids (17.3 ± 1.3 mg CAE/g). GC–MS profiling revealed a diverse set of compounds, including fat-soluble antioxidants like squalene, α-tocopherol, and γ-sitosterol, and water-soluble phenolics like pyrogallol and catechol, suggesting PSE is equipped with a multi-layered antioxidant defence. In the animal experiment, rats were given each pesticide for 30 days, with or without PSE. All four pesticides caused clear oxidative stress in the liver: glutathione (GSH), total antioxidant capacity (TAC), antioxidant enzymes activities dropped, while markers of lipid damage (MDA) and free radical activity (DPPH) rose. Co-administration of PSE significantly restored GSH, TAC and antioxidant enzymes levels and reduced MDA and residual DPPH values compared to pesticide-only groups; these parameters were statistically comparable to the controls (*p* > 0.05), indicating a substantial recovery of hepatic redox balance. Histopathological examination of liver tissues confirmed these findings, as pesticide treatment caused visible liver injury; deltamethrin and thiamethoxam led to congestion in central veins, while rotenone and acetochlor triggered clusters of inflammatory Kupffer cells. In animals that also received PSE, liver structure remained largely normal, with much less congestion and inflammation. These results show that the combination of antioxidant constituents in PSE might contribute to hepatoprotection through redox modulation and preservation of endogenous antioxidant balance, as suggested by the observed biochemical and histological improvements.

## 1. Introduction

Long-term, low-level exposure to pesticides is a widespread and serious public health issue worldwide, extending well beyond occupational settings to affect the general population through contaminated food, water, and air [1]. Epidemiological studies have reported associations between certain pesticide exposures and increased risk of selected cancers, including liver malignancies, although findings vary according to compound type, exposure level, and population characteristics [2]. The carcinogenicity of pesticides is not driven by a single mechanism but results from a multifaceted attack on cellular integrity. For instance, certain pesticides inhibit Phase I and Phase II detoxification enzymes in the liver, allowing toxic compounds to accumulate. They can also cause direct cell damage, activate pro-oncogenic signalling cascades such as MAPK and NF-κB, and disrupt cellular homeostasis, processes that collectively create conditions conducive to tumour development [1,2,3].

A key common thread in how pesticides cause harm is oxidative stress. Regardless of whether they are meant to kill insects, weeds, or fungi, many pesticides also act as strong pro-oxidants in mammals. Through processes such as redox cycling, metabolic activation, or direct damage to mitochondria, these chemicals cause a large and lasting spike in reactive oxygen species (ROS). This includes superoxide anions, hydrogen peroxide, and hydroxyl radicals [4,5]. This sudden buildup of ROS exceeds the body’s built-in antioxidant defences, including enzymes such as superoxide dismutase, catalase, and glutathione peroxidase, as well as small molecules like glutathione. Eventually, these defences become depleted.

Once the redox balance is disrupted, ROS can attack important cellular components directly. For example, they damage polyunsaturated fats in cell membranes, a process called lipid peroxidation that produces compounds like malondialdehyde (MDA). This weakens membrane structure and function. ROS also modify proteins through carbonylation, which can disable enzymes and interfere with cell signalling. Perhaps most seriously, oxidative damage to DNA in both the nucleus and mitochondria can cause breaks, altered bases, and mutations. If not repaired, these DNA changes are critical early steps in cancer development [6,7]. Given the serious cellular damage that ROS can cause, boosting the body’s natural antioxidant defences has become a key research goal, especially to counteract harm from pesticides. Medicinal plants offer a promising and culturally rich source of these protective compounds. Plants contain a diverse mix of bioactive molecules, flavonoids, phenolic acids, terpenoids, alkaloids, and lignans, among others. Together, these phytochemicals provide a layered defence. They can directly neutralise free radicals, bind metals to block harmful Fenton reactions, and even activate cellular antioxidant pathways like Keap1-Nrf2-ARE, which turns on the body’s own protective enzymes [8,9,10,11]. Beyond their immediate antioxidant role, these plant-derived compounds also serve as important starting points for developing new preventive and therapeutic drugs. This makes plants a sustainable, multi-target option for fighting chemically induced diseases.

Among the many plants known to have bioactive properties, *Pteropyrum scoparium* stands out as a promising candidate. Native to arid regions, this plant has already shown protective effects in studies of oxidative liver injury. For example, Al Nasiri et al. [12] found it helped to prevent liver damage in rats exposed to oxidised palm oil, and other work suggests it may also reduce colon cancer risk in experimental models [12,13,14]. These findings point to a phytochemical-rich profile capable of fighting oxidative stress, a good reason to study it further.

Still, a major gap remains in the research. While *P. scoparium* shows general antioxidant activity, its specific and comparative efficacy against oxidative stress triggered by a broad range of agriculturally relevant pesticides has not been investigated. Different pesticides cause harm in different ways: deltamethrin can disrupt calcium balance and trigger ROS, thiamethoxam impairs mitochondria, acetochlor generates toxic metabolites via liver enzymes, and rotenone blocks mitochondrial complex I. In real-world settings, people are often exposed to mixtures of pesticides, so finding a single natural extract that can protect against several types at once would be especially valuable.

Therefore, this study aims to evaluate the broad-spectrum hepatoprotective and antioxidant potential of *Pteropyrum scoparium* leaf extract (PSE) against a panel of chemically diverse pesticides. By doing so, it addresses a critical need in environmental toxicology and preventive medicine, with implications for developing natural, multi-target interventions against pesticide-induced liver damage.

## 2. Materials and Methods

### 2.1. Collection and Authentication of Plant Material

About 3 kg of fresh leaves of *the Pteropyrum scoparium* plant (accession No. 202100319) were collected from the Oman Botanic Garden in Muscat, Oman, in October 2023. Three separate samples were taken and were immediately washed with deionised water and kept at −20 °C for later use.

### 2.2. Preparation of Pteropyrum scoparium Plant Powder

Leaves of *Pteropyrum scoparium* plants were subjected to freeze-drying for five days at −40 °C using a freeze-drying system (LABCONCO, Kansas City, MO, USA). After this step, the samples were ground into powder using an electric grinder (Impex, Kerala, India) and the powdered material was stored in airtight plastic containers at −20 °C until processing.

**Figure 1:**
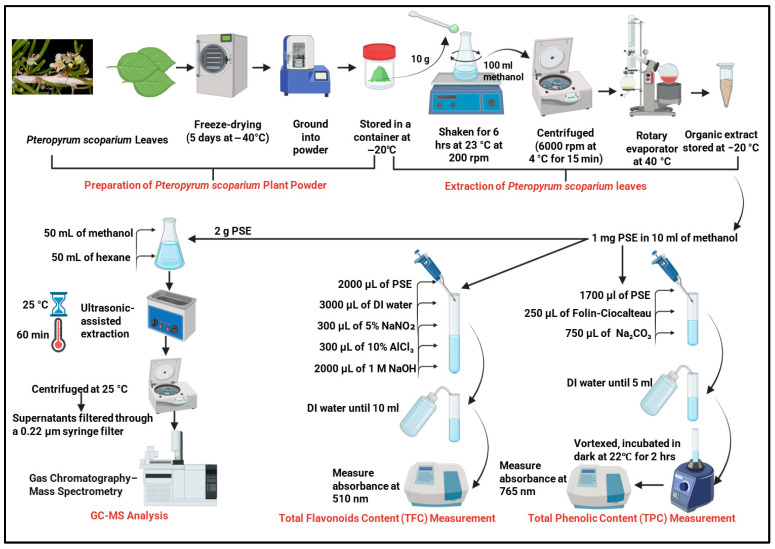
Schematic workflow of *Pteropyrum scoparium* leaf preparation, extraction and phytochemical analysis.

Figure 1 outlines the process for preparing, extracting, and analysing the *Pteropyrum scoparium* leaf extract.

### 2.3. Extraction of Pteropyrum scoparium Leaves

Organic extraction of the plant samples was prepared according to Al Nasiri et al. (2023) [12]. Briefly, 10 g fine powder from the freeze-dried plant sample was mixed with methanol (100 mL), and then the mixture was shaken for 6 h at room temperature (23 °C) at 200 rpm. The mixture was then centrifuged (6000 rpm/4 °C/15 min) using the Thermo Scientific Heraeus Megafuge 16R centrifuge (Thermo Electron LED GmbH Zweigniederlassung Osterode Am Kalkberg, 37520 Osterode am Harz, Germany). Methanol in the supernatant was removed using a rotary evaporator (G3 Heidolph, Schwabach, Germany) set at 40 °C. The obtained crude organic extract (50 g) was then stored at −20 °C until further analysis.

### 2.4. Total Phenolic Content (TPC) Measurement

The total phenolic content of the PSE was quantified using the well-established Folin–Ciocalteu colourimetric assay, which is based on the reduction of a phosphomolybdate–phosphotungstate complex by phenolic compounds, resulting in a blue chromophore measurable spectrophotometrically [15]. A stock solution of the crude PSE was prepared by dissolving 1.0 mg of the extract in 10.0 mL of methanol to achieve a concentration of 0.1 mg/mL. For the assay, 1.7 mL of this PSE solution was aliquoted into a series of 5 mL test tubes. To this, 250 µL of the Folin–Ciocalteu reagent was added, and the mixture was allowed to stand for 5 min to enable the initial redox reaction. Subsequently, 750 µL of a freshly prepared 1.9 M sodium carbonate (Na_2_CO_3_) solution was added to alkalinise the medium, which is necessary for the development of the final blue colour. The total reaction volume was adjusted to 5.0 mL by the addition of deionised water. The mixture was vortexed for 30 s to ensure homogeneity. The tubes were then kept in the dark at room temperature (about 22 °C) for 2 h so the colour could fully develop. Once the incubation was finished, we measured the absorbance of each sample at 765 nm. We used a reagent blank prepared the same way but with methanol instead of the plant extract as a reference and read the samples on a Multiskan Go Microplate Spectrophotometer (Thermo Fisher Scientific, Waltham, MA, USA). All measurements were performed in triplicate to ensure reliability.

A calibration curve was prepared using gallic acid as a reference standard. Known concentrations of gallic acid were prepared and processed following the same assay procedure. The absorbance values of the standards were plotted against their concentrations to generate a linear calibration curve. The total phenolic content of the PSE was then calculated by comparing the sample’s mean absorbance to this standard curve. The result was expressed as milligrams of Gallic Acid Equivalents per gram of dry plant extract (mg GAE/g), and data are presented as the means of three independent measurements ± standard deviation (SD).

### 2.5. Total Flavonoid Content (TFC) Measurement

The total flavonoid content of the PSE was determined using a colourimetric assay based on the formation of a stable yellow-orange complex between flavonoids and aluminium chloride [16]. An aliquot of the crude organic extract stock solution, prepared as described in Section 2.4 (1 mg extract in 10 mL methanol, 0.1 mg/mL), was used for the analysis. Precisely 2.0 mL of the PSE solution was transferred into a 10 mL volumetric test tube. To this, 3.0 mL of deionised water was added, followed by sequential additions of 300 µL of a 5% (*w*/*v*) sodium nitrite (NaNO_2_) solution and 300 µL of a 10% (*w*/*v*) aluminium chloride (AlCl_3_) solution. The mixture was vortexed briefly after each addition. The reaction was allowed to proceed for exactly 5 min at room temperature to ensure complete complex formation between the aluminium ions and the flavonoid catechol groups.

Following this incubation, 2.0 mL of a 1 M sodium hydroxide (NaOH) solution was added to the mixture, which immediately altered the solution colour. The total volume was then made up to 10.0 mL with deionised water. The final mixture was vortexed thoroughly for 30 s to ensure uniformity. The absorbance of the developed colour was measured at a wavelength of 510 nm against a reagent blank, prepared in an identical manner but with methanol replacing the PSE solution, using a Multiskan Go Microplate Spectrophotometer.

Quantification was achieved using a standard calibration curve prepared with catechin as the reference flavonoid. A series of catechin standard solutions across a known concentration range was processed using the same assay protocol. The absorbance values were plotted against catechin concentrations to generate a linear calibration curve. The total flavonoid content of the PSE was calculated by comparing the mean absorbance of the sample to this standard curve. The result is expressed as milligrams of Catechin Equivalents per gram of dry plant extract (mg CAE/g). All analyses were conducted in triplicate, and data are reported as the mean of these three independent measurements ± standard deviation (SD).

### 2.6. GC–MS Analysis of PSE

#### 2.6.1. Preparation of Extracts for GC–MS Analysis

Two grams (2 g) of the powdered PSE were mixed with 50 mL of methanol and 50 mL of hexane in a conical flask. The mixture was subjected to ultrasonic-assisted extraction using a sonicator to improve solvent penetration and compound dissolution. The ultrasonication was performed at a frequency of 40 kHz, a temperature of 25 °C, and for 60 min. After that, the samples were centrifuged at 25 °C, and the resulting supernatants were filtered through a 0.22 µm syringe filter [17].

#### 2.6.2. Instrumental Conditions for GC–MS Analysis

The methanol and hexane extracts were analysed using a Shimadzu GC-2010 Plus gas chromatograph (Shimadzu Corporation, Kyoto, Japan) coupled with a GC–MS-QP2010 Ultra mass spectrometer (Shimadzu Corporation, Kyoto, Japan). An AOC-20i auto-injector was used for automated sample introduction. Separation was performed on a polar fused-silica capillary column (SP-2560, Supelco, Bellefonte, PA, USA; 100 m × 0.25 mm internal diameter, 0.25 µm film thickness), which effectively separates fatty acids, sterols, and similar biological compounds.

High-purity helium gas (99.99%) served as the carrier gas at a constant linear flow rate of 1.0 mL/min. The injector port temperature was set at 250 °C. A 1.0 µL aliquot of each filtered extract was injected in split mode with a split ratio of 10:1. The GC oven temperature program was optimised as follows: initial temperature held at 42 °C for 1.3 min, then increased at a rate of 5 °C per minute to a final temperature of 300 °C, which was maintained for 10 min to ensure elution of high-boiling-point compounds. The total run time was approximately 63 min.

The transfer line connecting the GC to the MS was maintained at 240 °C, and the ion source temperature was set at 275 °C. Ionisation was carried out in electron impact (EI) mode at 70 eV. The mass spectrometer was operated in full-scan mode, scanning a mass-to-charge (*m*/*z*) range from 35 to 800 atomic mass units. The detector voltage (electron multiplier) was automatically calibrated using the instrument’s internal autotune procedure based on perfluorotributylamine (PFTBA). Compound identification was performed by comparing the mass spectra of the detected peaks with reference spectra in two major commercial libraries: the Wiley Registry of Mass Spectral Data (9th edition) and the National Institute of Standards and Technology (NIST version 2.3) mass spectral library. A match quality threshold of 85% or higher, combined with the comparison of calculated retention indices with published values where available, was used for the tentative identification of the phytochemical constituents.

### 2.7. Preparation and Administration of Pesticides

Four distinct pesticides, representing major classes of agrochemicals, were selected for this study to evaluate the broad-spectrum hepatoprotective potential of PSE. The pesticides included: acetochlor (CAS 34256-82-1, a chloroacetamide herbicide), deltamethrin (CAS 52918-63-5, a type II pyrethroid insecticide), rotenone (CAS 83-79-4, a botanical isoflavonoid insecticide and mitochondrial inhibitor), and thiamethoxam (CAS 153719-23-4, a neonicotinoid insecticide). All pesticides were procured as analytical standards of high purity (≥98%) from Molekula (Newton Aycliffe, UK) to ensure consistency and reliability in dosing.

Each pesticide was prepared individually for injection by dissolving 1.0 mg of the pure compound in 1.0 mL of pharmaceutical-grade corn oil. The mixture was gently vortexed and then sonicated in a water bath at 37 °C for 5 min to ensure complete dissolution and uniformity, with a fresh batch prepared daily. A dose of 1 mg/kg body weight per day was selected, based on the model established by El-Gohary et al. [18]. The selected dose (1 mg/kg/day for 30 days) was intentionally chosen as a sub-lethal, oxidative stress-inducing dose, rather than an acute toxic dose, to model progressive biochemical injury without overt hepatic failure. Importantly, this dose is substantially below reported LD50 values as follows: deltamethrin oral LD50 (rat): ~30–50 mg/kg, acetochlor oral LD50 (rat): >1000 mg/kg, thiamethoxam oral LD50 (rat): ~1500 mg/kg and rotenone oral LD50 (rat): ~132–150 mg/kg. Thus, 1 mg/kg represents a fraction of acute lethality thresholds and aligns with sub-chronic mechanistic toxicology studies, thereby allowing an evaluation of the protective effects of PSE during progressive injury. Corn oil served as the vehicle, and a separate vehicle control group confirmed that the oil itself did not influence the outcomes, while also providing an appropriate medium for administering these lipophilic compounds.

### 2.8. Experimental Animal Design and Treatment Protocol

**Figure 2:**
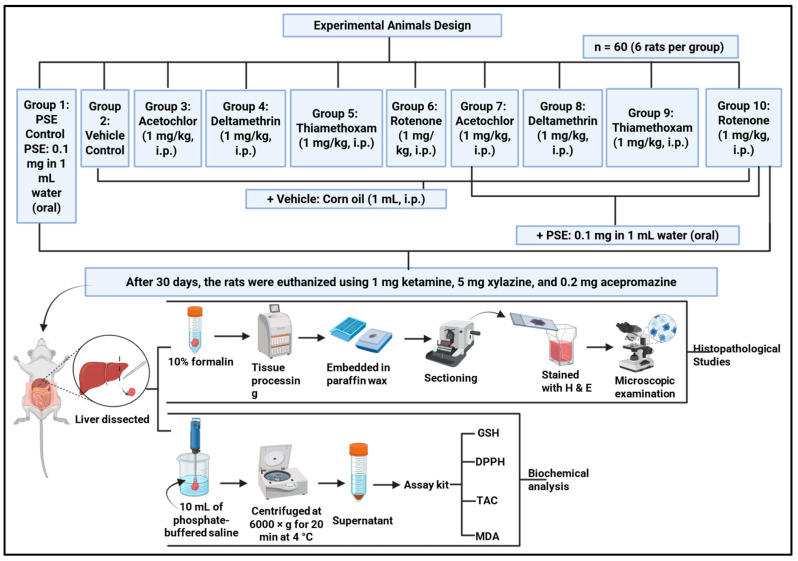
Overview of the experimental design.

Figure 2 outlines overall experimental design.

Sixty healthy adult male Sprague Dawley rats, approximately two months old and weighing 270 ± 5 g, were used in the study. All animals underwent a one-week acclimatisation period before experiments began. Throughout the study, animals were kept under controlled conditions: temperature was maintained at 22 ± 2 °C, humidity at 60 ± 5%, and a 12 h light/dark cycle (lights on at 07:00). They had free access to standard rodent pellets and filtered water. All procedures were approved by the Institutional Animal Ethics Committee and followed international guidelines for laboratory animal care.

After acclimatisation, rats were randomly assigned to ten groups of six animals each. Randomisation was carried out by computer to ensure body weights were evenly distributed and to avoid bias. The groups included a vehicle control (corn oil only), a PSE-only control, with four groups receiving one pesticide each and four groups receiving a pesticide together with PSE. The control group received a daily intraperitoneal injection of corn oil (1 mL/kg). The PSE control group was given oral *Pteropyrum scoparium* extract (0.1 mg/kg in 1 mL water) plus the corn oil injection. The selected oral PSE dose (0.1 mg/kg/day) represents a conventional literature-supported dose used in prior in vivo hepatoprotective and oxidative stress models, rather than a formally established minimal effective dose in the present experimental setting [12]. The aim of this study was to evaluate proof of concept hepatoprotection under a fixed dose design, rather than to conduct a full dose response analysis.

Pesticide groups were injected daily with acetochlor, deltamethrin, thiamethoxam, or rotenone at 1 mg/kg in corn oil, a dose representing chronic low-level exposure [18]. Corresponding intervention groups received the same pesticide injection along with oral PSE (0.1 mg/kg). Treatments continued for 30 days. Oral PSE (or its vehicle) was given in the morning, and pesticide (or vehicle) injections followed in the afternoon, with at least four hours between administrations to limit direct interactions. Health, behaviour, and body weight were checked weekly to monitor welfare and to help interpret any biochemical or tissue changes observed later. Body weight (gm) was recorded weekly for the entire duration of the experiment.

#### 2.8.1. Tissue Collection and Processing

After 30 days, the rats were euthanised using a lethal dose of a solution containing 1 mg ketamine, 5 mg xylazine, and 0.2 mg acepromazine. The liver tissue was dissected from each rat and divided into two parts: one part was preserved immediately in 10% formalin for histopathological evaluation, while the other part (2 g) was homogenised in 10 mL of phosphate-buffered saline (pH 7.4) with an IKA ULTRA TURRAX homogeniser (IKA-Werke GmbH & Co. KG, Staufen, Germany). The resulting rat liver homogenate was then centrifuged at 6000× *g* for 20 min at 4 °C using a CL30R Centrifuge (Staufen, Germany) and the supernatant was used for biochemical analysis.

#### 2.8.2. Biochemical Analysis of Oxidative Stress Markers

Before measuring oxidative stress markers, we determined the total protein content in each liver supernatant to normalise the data and account for differences in tissue amount or processing. Protein levels were measured using the Lowry method [19]. In brief, samples were mixed with an alkaline copper tartrate solution, followed by Folin–Ciocalteu reagent. The blue colour that developed was read at 750 nm. A standard curve was prepared with bovine serum albumin (0–1000 µg/mL) and sample protein concentrations were calculated from this curve.

Reduced glutathione (GSH), a key intracellular antioxidant, was measured with a commercial fluorometric kit (Sigma-Aldrich, CS1020, St. Louis, MO, USA). In this assay, GSH reacts specifically with o-phthalaldehyde under alkaline conditions to produce a fluorescent signal. In practice, deproteinised liver supernatant samples, prepared by precipitation with meta-phosphoric acid, were incubated with the OPT reagent. The fluorescence intensity was measured at an excitation wavelength of 340 nm and an emission wavelength of 420 nm using a microplate reader. The GSH concentration in each sample was determined by comparison to a concurrently run standard curve of reduced glutathione.

Total Antioxidant Capacity (TAC) was assessed using a colourimetric commercial kit (ELabscience, E-BC-K136-S, Houston, TX, USA). This assay employs the 2,2′-azino-bis (3-ethylbenzothiazoline-6-sulfonic acid) (ABTS) radical cation decolourisation method. The ABTS radical, which has a characteristic blue-green colour, is generated by oxidation of ABTS with potassium persulfate. Antioxidants in the liver tissue supernatant reduce the ABTS radical to its colourless form proportionally to their concentration. The decrease in absorbance was measured at 734 nm. Trolox, a water-soluble vitamin E analogue, was used as the standard, and the results are expressed as micromoles of Trolox equivalents per milligram of protein.

Lipid peroxidation, a key indicator of oxidative damage to membrane lipids, was evaluated by measuring the concentration of malondialdehyde (MDA), a primary thiobarbituric acid reactive substance (TBARS). The assay was performed using a specific commercial kit (Sigma-Aldrich, MAK568). In this method, MDA in the sample reacts with thiobarbituric acid (TBA) under acidic conditions at high temperature (95 °C) to form a pink MDA–TBA adduct. After cooling, the adduct was extracted with an organic solvent and its absorbance was measured spectrophotometrically at 532 nm. A standard curve was prepared using known concentrations of MDA standard provided with the kit, and the MDA concentration in the samples was calculated accordingly.

The DPPH, 2,2-diphenyl-1-picrylhydrazyl, assay was employed as an indirect measure of the overall radical scavenging capacity of liver homogenates, reflecting cumulative non-enzymatic antioxidant potential rather than endogenous DPPH presence in tissue. In this context, the assay provides complementary information to TAC and GSH measurements by assessing the ability of hepatic soluble components to reduce an exogenous stable radical under standardised conditions. The results are expressed as the percentage of DPPH radical scavenged relative to a control reaction containing no tissue extract.

The DNA was isolated from the liver tissue homogenates, and the DNA oxidative damage was assessed by measuring the formation of 8-hydroxydeoxyguanosine (8-OHdG) using the DNA Damage ELISA assay kit (OxiSelect™ Oxidative, catalogue number STA-320, Cell Biolabs Inc., San Diego, CA, USA); the quantity of 8-OHdG in the extracted DNA of each liver tissue (ng/mL) was measured at 450 nm and quantified by comparison with a 8-OHdG standard curve.

The antioxidant enzymes assay kits were purchased from Sigma-Aldrich, USA and were measured in accordance with the manufacturer’s instruction as follows: Catalase (CAT) with assay kit catalogue number CAT100, Glutathione Peroxidase (GPx) with assay kit catalogue number CGP1, and Superoxide Dismutase (SOD) with assay kit catalogue number CS0009.

#### 2.8.3. Histopathological Processing and Evaluation

Formalin-fixed liver samples were routinely processed. Samples were dehydrated through a series of graded ethanol solutions and cleared with xylene. The tissues were then embedded in paraffin wax, and sections of 5 μm thickness were cut using a rotary microtome and subsequently stained with hematoxylin and eosin. All prepared sections were examined at 200× magnifications using an Olympus BX51 microscope equipped with an Olympus DP70 camera (Hachioji, Tokyo, Japan).

For the scoring of liver congestion or inflammation, sections were assessed based on the following lesion score with slight modification. Score 0: no congestion or sinusoidal dilatation; score 1: mild congestion or centrilobular sinusoidal dilatation; score 2: moderate congestion or midzonal sinusoidal dilatation; score 3: severe congestion or periportal sinusoidal dilatation. For the severity of inflammation scoring, 0 = no or few inflammatory cells; 1 = mild hepatic inflammation or periportal inflammation; 2 = moderate hepatic inflammation or periportal and intraparenchymal inflammation; and 3 = severe hepatic inflammation with hepatic necrosis. Three randomly selected 40× microscopic fields were examined from each liver section of every rat across the ten groups, and the score was determined based on the mean percentage.

### 2.9. Statistical Analysis

The results are presented as mean ± standard deviation (SD). Statistical analysis was conducted using one-way analysis of variance (ANOVA) followed by Tukey’s test, using GraphPad Prism (GraphPad Prism 10.1.2 Software Inc., San Diego, CA, USA). A *p* value of less than 0.05 is considered statistically significant.

## 3. Results and Discussion

### 3.1. Phytochemical Analysis of Phenolic and Flavonoid Content

This indicates that the PSE extract possesses substantial reducing capacity, as measured by the Folin–Ciocalteu assay, which reflects the presence of redox-active constituents. The Folin–Ciocalteu assay indicated that the methanolic PSE exhibited a high total reducing capacity, expressed as TPC of 345.1 ± 7.6 mg gallic acid equivalents per gram of dried extract. The aluminium chloride assay, which is specific for flavonoids, gave a TFC of 17.3 ± 1.3 mg of catechin equivalents per gram. Although flavonoids constitute a subclass of phenolic compounds, the lower total flavonoid content (TFC) relative to the total phenolic content (TPC) indicates that non-flavonoid phenolics represent a substantial proportion of the extract’s phenolic fraction. GC–MS analysis further identified several non-flavonoid phenolic compounds, including simple phenolic acids and pyrogallol derivatives.

The high TPC result puts PSE among plant extracts with strong antioxidant potential. This agrees with earlier work on Omani plants; for example, Al Saidi et al. [13] reported that an aqueous extract of *P. scoparium* also had high phenolics and antioxidant capacity compared to other local species. Our methanolic extract’s TPC supports the finding and suggests methanol is an effective solution for pulling out these compounds.

Even though the flavonoid content is lower, the 17.3 mg/g of flavonoids still matters. Flavonoids are known to fight oxidative stress in several ways: they scavenge free radicals directly, bind metal ions, and can even help recycle other antioxidants like α-tocopherol [8,11]. They often work together with other phenolics and their structure lets them interact with cell membranes and proteins, adding another layer of protection.

### 3.2. GC–MS Phytochemical Profiling of PSE

**Figure 3:**
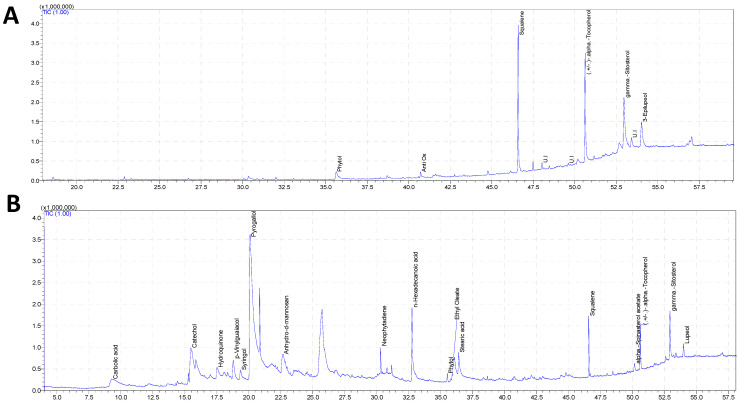
Total ion chromatogram for (**A**) the hexane extract and (**B**) the methanol extract of PSE.

The *Pteropyrum scoparium* leaf extract (PSE) was analysed using GC–MS with two solvents: hexane (for fat-soluble compounds) and methanol (for water-soluble ones). The methanol extract showed a more complex mix of polar and semi-polar molecules (Figure 3B). Seventeen major components were identified (Table 1), with most of them being phenolics. The largest peak was pyrogallol (34.92%), a simple polyphenol with three hydroxyl groups that make it a powerful reducing agent and radical scavenger [20,21]. Other phenolics like catechol (5.74%) and hydroquinone (2.17%) added to this scavenging ability. Compounds containing the catechol structure are known to inhibit stress-sensitive signalling pathways, such as NF-κB, which help reduce inflammation [22].

Besides phenolics, the methanol extract also contained fatty acids such as palmitic acid (5.86%) and stearic acid (2.11%), which might affect how other compounds are absorbed or how cells use them. The methanol also pulled out some of the same fat-soluble compounds found in the hexane fractions, squalene (2.53%), γ-sitosterol (2.97%), and α-tocopherol (2.12%), showing that the crude PSE naturally contains both water-soluble and fat-soluble protective compounds. Lupeol (0.85%) was also found in the methanol extract, which has anti-inflammatory and anti-apoptotic properties [23].

The hexane extract contained mainly large, fat-soluble molecules, which showed up later in the chromatogram (retention time 35–54 min; Figure 3A). Seven major compounds were found, and three related terpenoids made up over 78% of the total (Table 2). The most abundant was squalene (28.11%), a linear triterpene that not only helps make cholesterol but also acts as a strong antioxidant, especially against singlet oxygen [24,25]. Next was (±)-α-tocopherol (27.45%), the main form of vitamin E and a chain-breaking antioxidant that halts lipid peroxidation in cell membranes [25]. The third major component was γ-sitosterol (22.57%), a plant sterol that can blend into cell membranes and may make them more resistant to oxidative damage [25]. Other noteworthy compounds in the hexane fraction were 3-epilupeol (10.90%), a triterpenoid with anti-inflammatory and anti-apoptotic effects [24] and phytol (4.14%), a diterpene from chlorophyll that also helps produce tocopherols [24].

The different chemical profiles of the two extracts highlight how solvent choice shapes what gets extracted. Hexane extracts nonpolar terpenoids and sterols, while methanol grabs polar phenolics and acids. The water-soluble phenolics likely neutralise ROS in the cell’s interior, while the fat-soluble compounds embed themselves in cell membranes, stabilising them and quenching lipid-soluble radicals.

Although GC–MS profiling identified several bioactive constituents within PSE, including pyrogallol, catechol, lupeol, and γ-sitosterol, the present findings should not be interpreted as evidence that any single compound independently mediates the observed hepatoprotective effects. At the administered dose of 0.1 mg/kg body weight/day, the estimated theoretical exposure to individual constituents based on relative peak area percentages would fall within the microgram per kilogram range. These values are considerably lower than doses commonly used in isolated compound pharmacological studies.

Low molecular weight phenolics such as pyrogallol and catechol are known to undergo rapid phase II metabolism; however, accumulating evidence indicates that dietary polyphenols may exert biological effects at low micromolar concentrations primarily through the modulation of redox-sensitive signalling pathways, including Nrf2 and NF-κB, rather than through direct stoichiometric radical scavenging alone [26,27]. Similarly, phytosterols and triterpenoids such as γ-sitosterol and lupeol demonstrate membrane incorporation and anti-inflammatory signalling modulation in vivo, even at modest exposure levels, particularly within complex plant matrices [10,25]. Therefore, the hepatoprotective effects observed in this study are more appropriately attributed to the integrated and potentially synergistic action of the phytochemical matrix rather than to isolated high-dose pharmacological activity of individual compounds. This concept is well supported in the polyphenol literature, where low systemic concentrations still result in measurable modulation of cellular antioxidant defences [28,29].

### 3.3. Body Weight Progression in Rats During the 30-Day Treatment Period

**Figure 4:**
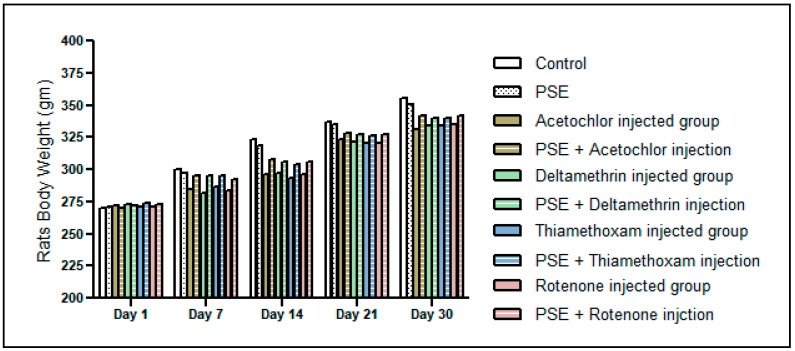
Body weight progression in rats during the 30-day treatment period. Data are presented as mean ± SD (n = 6 per group). Body weights were measured at baseline (day 1) and weekly thereafter (days 7, 14, 21, and 30). No statistically significant differences in weight gain were observed between treatment and control groups (*p* > 0.05), indicating absence of overt systemic toxicity.

As presented in Figure 4, all experimental groups demonstrated progressive body weight gain in comparison to the control group, where *p* > 0.05. The absence of significant weight suppression suggests that the selected dose (1 mg/kg/day) induced sub-clinical oxidative stress without causing overt systemic toxicity, anorexia, or metabolic wasting. In toxicological models, marked body weight reduction reflects severe hepatic dysfunction, and in our study, the preservation of normal growth indicates that the administered pesticide doses were sufficient to induce measurable redox imbalance at the biochemical level, as evidenced by altered TAC, GSH, MDA, and DPPH parameters, but did not disrupt whole body energy homeostasis. Furthermore, co-administration of *Pteropyrum scoparium* extract (PSE) did not impair normal weight gain, supporting its safety and tolerability at 0.1 mg/kg. Collectively, these findings reinforce that the hepatoprotective effects observed in subsequent analyses are unlikely to be secondary to confounding differences in nutritional status or systemic toxicity but rather reflect the targeted modulation of oxidative pathways within liver tissue.

#### 3.3.1. Measurement of Total Antioxidant Capacity and Glutathione Levels

##### Total Antioxidant Capacity (TAC)

**Figure 5:**
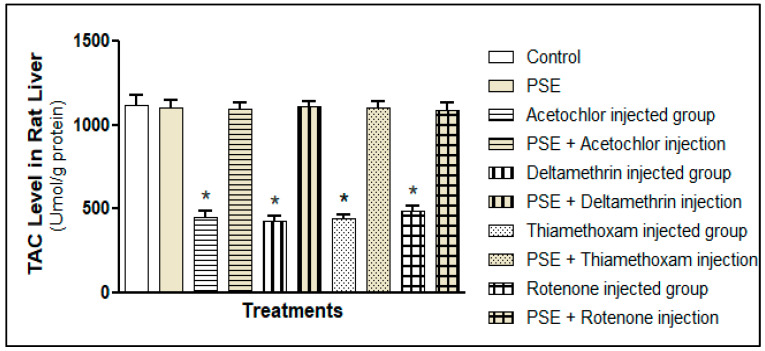
Total antioxidant capacity (TAC) level in rat liver tissue homogenates fed with PSE in the presence or absence of pesticide (acetochlor, deltamethrin, thiamethoxam, and rotenone) injections. * Significantly lower as compared to the control group, *p* < 0.05. Values without superscript are not significantly different compared to the control group, *p* > 0.05.

Each pesticide, acetochlor, deltamethrin, thiamethoxam, and rotenone, noticeably weakened the liver’s overall antioxidant defence. As shown in Figure 5, TAC levels dropped significantly in all the groups exposed to pesticides alone compared to the controls (*p* < 0.05). TAC reflects the combined strength of the liver’s antioxidants, including enzymes like superoxide dismutase and catalase, and small molecules such as uric acid, bilirubin, and glutathione. The drop in TAC means the liver’s ability to handle oxidative stress was worn down, leaving cells open to damage; a common result when pesticides produce ROS faster than the body can rebuild its defences [6,7]. Even though the four pesticides work differently, they all lowered TAC to a similar degree (*p* > 0.05), pointing to oxidative stress as a common harmful outcome.

Importantly, when PSE was given together with each pesticide, TAC returned to normal levels in these groups and matched those of healthy controls (*p* > 0.05; Figure 4). This recovery suggests PSE acts not only as a simple antioxidant; it might indirectly influence endogenous antioxidant balance. Phenolics in PSE, especially pyrogallol and catechol, likely donate electrons to directly neutralise ROS and may protect the liver’s built-in antioxidants from depletion [11].

##### Glutathione (GSH)

**Figure 6:**
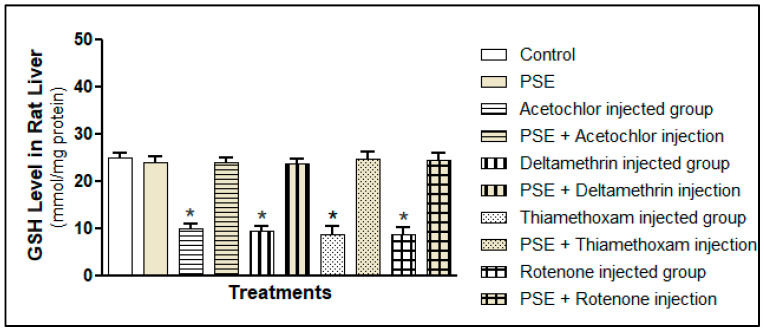
Total glutathione (GSH) level in rat liver tissue homogenates fed with PSE in the presence or absence of pesticide (acetochlor, deltamethrin, thiamethoxam, and rotenone) injections. * Significantly lower as compared to the control group, *p* < 0.05. Values without superscript are not significantly different compared to the control group, *p* > 0.05.

In parallel to the global TAC measurement, we quantified hepatic reduced glutathione (GSH), the central component of the thiol-based antioxidant system. GSH is a tripeptide (γ-glutamyl-cysteinyl-glycine) that serves as a critical intracellular reductant, a cofactor for antioxidant enzymes like glutathione peroxidase (GPx) and glutathione S-transferase (GST), and a direct scavenger of free radicals and electrophilic toxicants. As shown in Figure 6, exposure to each pesticide alone induced a significant depletion of hepatic GSH stores compared to the control (*p* < 0.05). This drop can be explained by how pesticides are processed: for example, deltamethrin and acetochlor are broken down into reactive intermediates that bind directly to GSH, a detoxification step that consumes GSH reserves, especially when pesticide exposure is high [30,31,32,33]. Rotenone also depletes GSH in a different way: by blocking mitochondrial complex I, it triggers a surge of superoxide that oxidises GSH to its inactive form (GSSG), shrinking the pool of active antioxidant glutathione [34]. Thiamethoxam has similarly been shown to induce oxidative stress that depletes GSH and impairs glutathione-related enzymes [35,36]. As with TAC, the extent of GSH loss was similar across all pesticide groups (*p* > 0.05), underscoring that oxidative stress is the common harmful outcome regardless of the pesticide’s initial mechanism.

The concurrent supplementation with PSE completely prevented this pesticide-induced GSH depletion. The GSH levels in all groups receiving both a pesticide and PSE were not statistically different from those in the control or PSE-only groups (*p* > 0.05) (Figure 5). This profound protective effect on the GSH system can be attributed to several synergistic actions of PSE phytochemicals. First, the direct antioxidant action of phenolic compounds reduces the oxidative burden, decreasing the rate of GSH consumption through non-enzymatic radical scavenging and lessening the demand on the GPx system to reduce peroxides. Second, flavonoids and other phenolics may contribute to maintaining redox balance, potentially supporting the GSH/GSSG cycle. Third, key compounds identified in PSE, such as γ-sitosterol and lupeol, have been shown in other models to enhance the activities of GSH-synthesising enzymes (γ-glutamylcysteine ligase) and GSH-recycling enzymes (glutathione reductase). By preserving the intracellular GSH concentration, PSE helps maintain essential cellular functions, including the detoxification of electrophiles and regulation of protein thiol status.

#### 3.3.2. Measurement of Lipid Peroxidation and Free Radical Scavenging Activity

##### Lipid Peroxidation (MDA)

**Figure 7:**
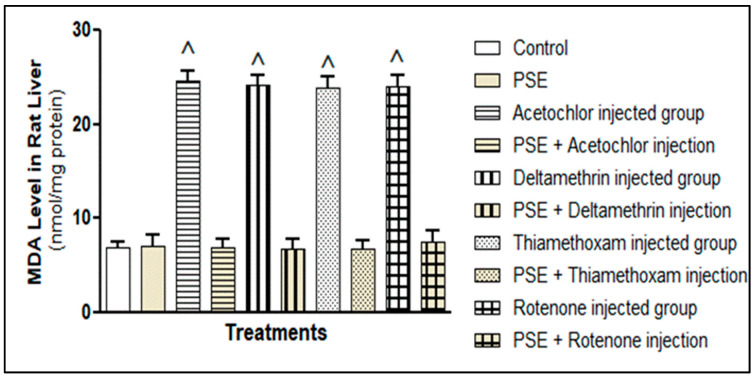
Lipid peroxidation as determined by malondialdehyde (MDA) in rat liver tissue homogenates fed with PSE in the presence or absence of pesticide (acetochlor, deltamethrin, thiamethoxam, and rotenone) injections. ^ Significantly higher as compared to the control group, *p* < 0.05. Values without superscript are not significantly different compared to the control group, *p* > 0.05.

Figure 7 shows that all four pesticides, acetochlor, deltamethrin, thiamethoxam, and rotenone, significantly raised liver MDA levels compared to the control group (*p* < 0.05). This increase reflects considerable oxidative damage to cell membranes, matching how each pesticide promotes oxidation. Deltamethrin triggers ROS via cytochrome P450 metabolism [37,38], thiamethoxam disrupts mitochondrial function [36,39], rotenone boosts superoxide by inhibiting complex I [34], and acetochlor generates reactive metabolites that start lipid peroxidation [31,40,41]. Although these pesticides act through different primary mechanisms, MDA elevation did not differ significantly among them (*p* > 0.05), underscoring lipid peroxidation as a common downstream result of oxidative stress.

When PSE was given together with each pesticide, membrane damage was sharply reduced. MDA levels in the co-treatment groups returned to near-normal, matching the control values (*p* > 0.05). This protection likely comes from the fat-soluble antioxidants in PSE, particularly α-tocopherol and squalene, which integrate into cell membranes and act as chain-breaking antioxidants. α-Tocopherol donates hydrogen to lipid radicals, halting the peroxidation chain, while squalene neutralises singlet oxygen and peroxyl radicals, thereby lowering MDA formation [25]. In addition, by maintaining GSH levels, PSE supports glutathione peroxidase activity, which converts lipid hydroperoxides into harmless alcohols, further limiting MDA generation.

##### Free Radical Scavenging Activity (DPPH)

**Figure 8:**
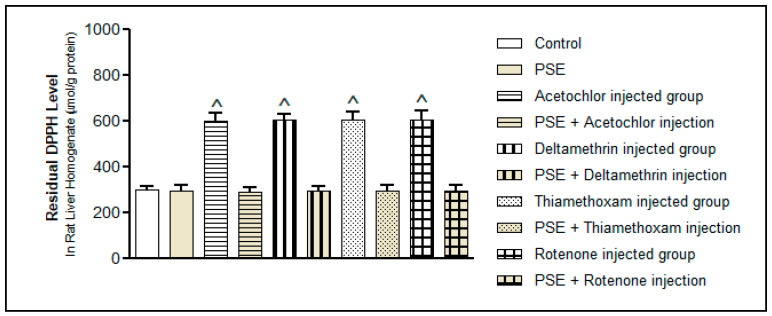
Residual 2,2-diphenyl-1-picrylhydrazyl DPPH level after reaction with rat liver homogenates with PSE in the presence or absence of pesticide (acetochlor, deltamethrin, thiamethoxam, and rotenone) injections. ^ Significantly higher as compared to the control group, *p* < 0.05. Values without superscript are not significantly different compared to the control group, *p* > 0.05.

The DPPH test shows how well the liver tissue can still neutralise free radicals. As Figure 8 illustrates, all pesticides increased leftover DPPH activity (*p* < 0.05), meaning the liver’s built-in scavenging ability was worn down either because antioxidants were used up or due to excessive radical production. This fits with the earlier drops in TAC and GSH, together confirming a system-wide redox imbalance. Like the other markers, the rise in DPPH activity was similar across pesticide groups (*p* > 0.05), again pointing to oxidative stress as the common problem.

Strikingly, giving PSE alongside each pesticide brought DPPH activity back to normal, matching control levels. This return to baseline highlights PSE’s strong direct radical-scavenging power, which comes mainly from its high phenolic content. Pyrogallol and catechol, major components in the methanol extract, are especially good at donating hydrogen or electrons to stabilise free radicals, thanks to their multiple hydroxyl groups [20,21]. Flavonoids in PSE also help by using their ring structures to trap radicals and by binding transition metals, which stops Fenton reactions that would otherwise produce more ROS [8].

#### 3.3.3. DNA Oxidative Damage

**Figure 9:**
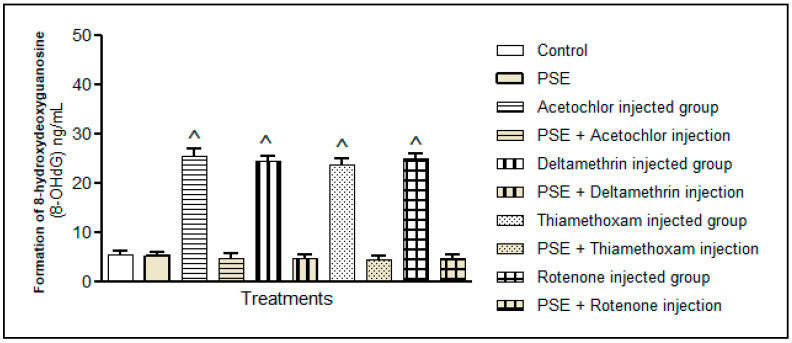
DNA oxidative damage. PSE extract supplementation showed a significant reduction in the hepatic oxidative DNA damage associated with pesticide (acetochlor, deltamethrin, thiamethoxam, or rotenone) injection. ^ Significantly higher as compared to the control group, *p* < 0.05. Values without superscript are not significantly different compared to the control group.

Among numerous types of oxidative DNA damage, the formation of 8-hydroxydeoxyguanosine (8-OHdG) is a ubiquitous marker of oxidative stress and the quantity of 8-OHdG (ng/mL) in the extracted DNA of each liver tissue was measured. Figure 9 revealed that the administration of each pesticide (acetochlor, deltamethrin, thiamethoxam, or rotenone) significantly elevated oxidative DNA damage in liver tissues compared to the control group, *p* < 0.05. This finding confirms the oxidative–genotoxic impact of pesticides, which generates reactive oxygen species capable of oxidising DNA bases and initiating mutagenic processes. Supplementation with the PSE substantially reduced the observed pesticide-associated DNA damage, where *p* < 0.05. The PSE protective mediated effect might be attributed to the antioxidant properties of polyphenols present in PSE, which can scavenge reactive oxidants before they interact with DNA. Additionally, supplementation of the PSE might maintain genomic integrity and enhance cellular DNA repair pathways, a mechanism documented for polyphenol-rich plant extracts. Therefore, PSE has the potential to attenuate oxidative DNA damage associated with pesticide exposure.

#### 3.3.4. Antioxidant Enzyme Activities

Exposure to acetochlor, deltamethrin, thiamethoxam, or rotenone resulted in a significant reduction in the activity of key antioxidant enzymes in the liver tissue, including superoxide dismutase (SOD), catalase (CAT), and glutathione peroxidase (GPx) (Table 3). Specifically, CAT, GPx and SOD enzyme activities in the pesticide groups were significantly lower than the control group (*p* < 0.05). The observed CAT, GPx and SOD decline in the assayed liver tissue reflects a compromised endogenous antioxidant system, thereby increasing susceptibility to hepatic oxidative injury. Administration of the PSE to the pesticide groups notably restored CAT, GPx and SOD enzyme activities (Table 1). These results demonstrate that the PSE treatment contributed to the reactivation of the liver’s intrinsic antioxidant defences.

Functionally, these antioxidant enzymes operate synergistically, as SOD converts superoxide radicals to hydrogen peroxide, which is subsequently metabolised by CAT and the coupled GPx system utilising glutathione. By strengthening this coordinated enzymatic cascade, PSE enhances the liver’s capacity to neutralise reactive oxygen species generated by pesticide exposure, thereby protecting hepatic cellular macromolecules such as lipids, proteins, and DNA from oxidative damage. Together, these results support the conclusion that PSE not only provides exogenous antioxidant compounds but also up-regulates the host’s endogenous enzymatic defence mechanisms.

### 3.4. Histopathological Correlation of Oxidative Injury and Phytotherapeutic Intervention

#### 3.4.1. Normal Liver Structure in Control and PSE-Only Groups

**Figure 10:**
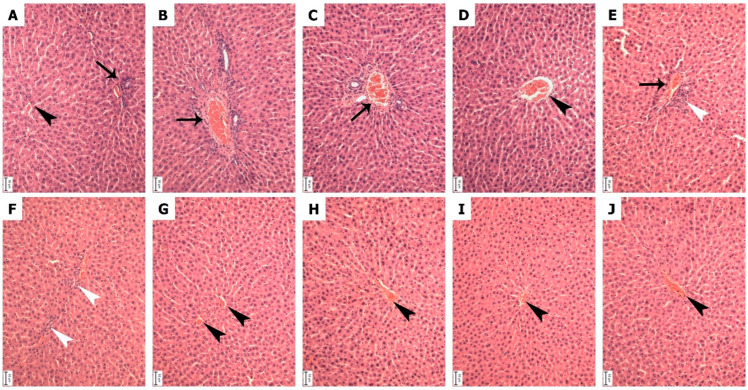
Histopathological examination of hepatocytes of rats treated with pesticides alone and with PSE. (Arrow = portal area; black arrowhead = central vein; white arrowhead = Kupffer cells). Control group (**A**), PSE group (**B**), Deltamethrin injected group (**C**) Thiamethoxam injected group (**D**) Rotenone injected group (**E**), Acetochlor injected group (**F**), PSE + Deltamethrin group (**G**), PSE + Thiamethoxam group (**H**), PSE + rotenone group (**I**), and PSE + acetochlor group (**J**). Scale bars: 50 μm.

Liver sections from the control group (A) and the group receiving PSE alone (B) showed healthy, typical tissue architecture (Figure 10). Hepatocytes were arranged in regular cords radiating from central veins, with intact cytoplasm and nuclei, and sinusoids showed normal patency. No pathological changes were observed in the PSE-only group, confirming that the extract was well-tolerated at the administered dose and establishing a reliable baseline for assessing pesticide-induced damage.

#### 3.4.2. Pesticide-Specific Patterns of Histological Injury

##### Vascular Congestion in Deltamethrin- and Thiamethoxam-Exposed Groups

Exposure to deltamethrin (group C) or thiamethoxam (group D) led primarily to hemodynamic disturbance, marked by significant congestion of the central veins (Figure 10). Liver-cell structure remained largely intact, suggesting early vascular damage preceding parenchymal injury. This finding aligns with reports that deltamethrin-induced oxidative stress disrupts vascular endothelial function and increases permeability, leading to sinusoidal and central venous congestion [37,42,43]. Similarly, thiamethoxam promotes oxidative damage to the endothelium, resulting in haemorrhage and the congestion of hepatic central veins [39,44]. The common vascular pathology across these two insecticides highlights how oxidative stress can first manifest as circulatory dysfunction within the liver.

##### Inflammatory Response in Rotenone- and Acetochlor-Exposed Groups

In contrast, rotenone (group E) and acetochlor (group F) provoked a more aggressive inflammatory reaction (Figure 10). The hallmark finding was prominent activation and aggregation of Kupffer cells within portal tracts and perivascular regions. As resident liver macrophages, Kupffer cell clustering signals a sustained inflammatory response to tissue injury. Rotenone, a mitochondrial complex I inhibitor, triggers profound intracellular oxidative stress and the release of pro-inflammatory mediators that recruit and activate Kupffer cells [34]. Acetochlor generates reactive metabolites that directly damage hepatocytes, initiating apoptosis and secondary inflammation mediated by Kupffer cells [31]. These aggregates reflect an escalation from oxidative stress to active immune-driven tissue inflammation.

#### 3.4.3. PSE-Mediated Attenuation of Histopathological Damage

##### Protection Against Vascular Congestion

Co-treatment with PSE in the deltamethrin (group G)- and thiamethoxam (group H)-exposed rats preserved normal central vein morphology, with no evidence of congestion (Figure 10). This suggests that PSE’s antioxidant components protected the vascular endothelium from oxidative insult, maintaining vascular integrity and preventing the hemodynamic stasis typical of these pesticide toxicities.

##### Reduction of Inflammatory Pathology

In the rotenone (group I)- and acetochlor (group J)-exposed groups, PSE co-administration dramatically reduced inflammatory infiltrates (Figure 10). Prominent periportal and perivascular Kupffer-cell aggregates were absent, and hepatic lobules maintained near-normal architecture. This anti-inflammatory effect is likely linked to specific PSE constituents such as lupeol and γ-sitosterol, which are known to inhibit the NF-κB signalling pathway, a central regulator of cytokine production and Kupffer-cell activation [23,25]. By dampening this cascade, PSE limits the amplification of tissue damage initiated by oxidative stress. Furthermore, PSE’s antioxidant phenolics and tocopherols may lower the initial oxidative signal that triggers inflammation.

#### 3.4.4. Integrated Histoprotective Mechanism of PSE

The broad-spectrum protection offered by PSE, against both vascular congestion (groups G, H) and immune-cell infiltration (groups I, J), underscores its multimodal mechanism (Figure 10). PSE is considered a radical scavenger and modulates cellular injury responses. The extract appears to intervene at multiple stages of the pesticide toxicity cascade: neutralising initiating ROS, protecting cellular and vascular structures, and suppressing downstream inflammation. This integrated action spanning antioxidant, membrane-stabilising, and anti-inflammatory activities ensures the structural and functional preservation of hepatic tissue. The histopathological data thus provide compelling visual confirmation that PSE functions as a potent antioxidant, capable of maintaining liver morphology.

The histopathological assessment of liver tissues demonstrated distinct group-related variations in lesion scores for congestion and inflammation is presented in Table 4. Groups A, G, H, I, and J exhibited no observable hepatic lesions, with both congestion and inflammation scores recorded as 0, indicating preserved liver architecture and an absence of detectable pathological alterations. In contrast, groups B, C, and D showed mild hepatic congestion (score 1) without accompanying inflammatory changes (score 0), suggesting limited vascular disturbance without progression to an inflammatory response. Conversely, groups E and F displayed mild inflammatory infiltration (score 1) in the absence of congestion (score 0), reflecting localised inflammatory activity independent of vascular engorgement. Overall, the findings indicate that hepatic lesions were mild and group-specific, with no evidence of concurrent congestion and inflammation in any experimental group.

## 4. Conclusions

The hepatoprotective effect of PSE may be interpreted as the result of complementary antioxidant layers operating within distinct cellular compartments. Lipophilic constituents such as α-tocopherol, squalene, and γ-sitosterol likely contribute to membrane-level protection by interrupting lipid peroxidation and stabilising phospholipid bilayers, consistent with the observed reduction in hepatic MDA. In parallel, low molecular weight phenolics including pyrogallol and catechol may function within aqueous intracellular environments, contributing to redox modulation and overall radical scavenging capacity, as reflected in the restored TAC and DPPH parameters. Preservation of hepatic GSH further indicates maintenance of endogenous glutathione-dependent defences, suggesting that PSE supports redox homeostasis primarily through attenuation of oxidative burden rather than direct substitution for enzymatic antioxidant systems. Collectively, these findings indicate that PSE-mediated hepatoprotection is best understood as a coordinated interaction between membrane stabilisation, cytosolic redox modulation, and the preservation of endogenous redox balance and glutathione-dependent defences.

Collectively, these findings suggest that PSE-mediated hepatoprotection is best understood as a coordinated interplay between membrane stabilization, cytosolic redox modulation, and the maintenance of endogenous redox homeostasis, including glutathione-dependent defense mechanisms.

In summary, the 1 mg/kg/day dosing regimen represents a validated sub-chronic oxidative stress; while it does not replicate precise environmental dietary intake levels, it reflects mechanistically relevant chronic systemic exposure and allows the reproducible evaluation of hepatoprotective interventions. In our study model, *Pteropyrum scoparium* extract acts a multi-target botanical agent that defends the liver against different types of pesticide-induced oxidative injury. These findings support the notion that *Pteropyrum scoparium* extract might act as a natural supplement with antioxidant potential properties against the aetiology of environmental toxin-mediated liver diseases.

Histopathological evaluation confirmed these results. Different pesticides, acetochlor, deltamethrin, thiamethoxam, and rotenone caused clear damage, vascular congestion with deltamethrin and thiamethoxam, and inflammatory Kupffer-cell clusters with rotenone and acetochlor. When PSE was added, the liver structure remained largely normal: congestion was reduced, and inflammation was significantly lower. This shows PSE not only acts as an antioxidant, but also helps protect blood vessels and dampens immune-driven inflammation.

## 5. Limitations of the Study

In this work, the direct measurement of pesticide plasma or hepatic concentrations was not performed in the present study. Future studies will include toxic kinetic profiling (LC–MS/MS quantification in plasma and liver tissue) to directly correlate the administered dose with internal concentrations.

## Figures and Tables

**Table 1:** GC–MS profile of the methanol extract of PSE.

S.No.	R.Time (min)	Area	Area%	Name	K.I (NiST)
1	9.30	6,617,751	4.02	Carbolic acid	981
2	15.48	9,452,760	5.74	Catechol	1197
3	17.51	3,576,818	2.17	Hydroquinone	1283
4	18.79	2,606,128	1.58	p-Vinylguaiacol	1315
5	19.35	2,035,848	1.24	Syringol	1316
6	20.10	57,471,844	34.92	Pyrogallol	1385
7	22.66	8,688,260	5.28	Anhydro-d-mannosan	1480
8	30.32	1,743,274	1.06	Neophytadiene	1840
9	32.76	9,637,026	5.86	n-Hexadecanoic acid	1942
10	35.53	689,279	0.42	Phytol	2114
11	36.08	4,264,487	2.59	Ethyl Oleate	2171
12	36.41	3,466,074	2.11	Stearic acid	2172
13	46.59	4,156,180	2.53	Squalene	2827
14	50.17	810,020	0.49	alpha.Spinasterol acetate	3429
15	50.63	3,480,848	2.11	(.+/-.)-.alpha.-Tocopherol	3149
16	52.94	4,886,403	2.97	gamma.-Sitosterol	3351
17	54.00	1,395,701	0.85	Lupeol	3399

**Table 2:** GC–MS profile of the hexane extract of PSE.

S.No.	R.Time (min)	Area	Area%	Name	K.I (NiST)
1	35.64	1,753,808	4.14	Phytol	2114
2	40.72	415,674	0.98	Anti Ox	2414
3	46.60	11,919,637	28.11	Squalene	2827
4	49.62	298,685	0.70	gamma.-Tocopherol	3074
5	50.63	11,643,560	27.45	(.+/-.)-.alpha.-Tocopherol	3149
6	52.97	9,573,658	22.57	gamma.-Sitosterol	3351
7	54.02	4,622,323	10.89	3-Epilupeol	3381

**Table 3:** Effect of PSE on antioxidant enzyme levels in pesticide-induced oxidative stress in rat liver.

	Group	Control	PSE	Acetochlor	PSE + Acetochlor	Deltamethrin	PSE + Deltamethrin	Thiamethoxam	PSE + Thiamethoxam	Rotenone	PSE + Rotenone
Enzymes											
Catalase (µmol/mg protein/min)											
1.69 ± 0.26			1.63 ± 0.14	0.57 ± 0.08 *	1.49 ± 0.17	0.55 ± 0.05 *	1.55 ± 0.22	0.56 ± 0.09 *	1.56 ± 0.19	0.54 ± 0.01 *	1.55 ± 0.28
Superoxide dismutase (units/mg protein)											
87.11 ± 0.81			86.95 ± 0.92	48.58± 0.55 *	86.45 ± 0.64	48.77 ± 0.41 *	85.92 ± 0.95	49.87 ± 0.73 *	86.58 ± 0.84	48.72 ± 0.86 *	86.13 ± 0.58
Glutathione peroxidase (µmol/mg protein/min)											
26.11 ± 1.01			25.94 ± 0.99	10.24 ± 0.64 *	25.89 ± 0.36	9.89 ± 0.50 *	25.84 ± 0.47	10.14 ± 0.58 *	25.91 ± 0.72	9.99 ± 0.47 *	25.87 ± 0.52

Each value represents mean ± SD of three determinations. * Significantly lower as compared to control group, *p* < 0.05. Values without superscript are not significantly different as compared to control group, *p* > 0.05.

**Table 4:** Lesion scores for liver congestion or inflammation of different groups.

Groups	Congestion Score	Inflammation Score
A, G, H, I, J	0	0
B, C, D	1	0
E, F	0	1

## Data Availability

The original contributions presented in this study are included in the article. Further inquiries can be directed to the corresponding authors.
